# *In Silico* Analysis and Transcriptional Profiling of A Putative Metalloprotease ADAMTSL as A Potential Tick Antigen against *Rhipicephalus microplus*

**DOI:** 10.3390/pathogens14020190

**Published:** 2025-02-14

**Authors:** Cesar Onoshi Sedano-Juarez, Ninnet Gómez-Romero, Miguel Ángel Alonso-Díaz, América Ivette Barrera-Molina, David Emanuel Reyes-Guerrero, Rodolfo Lagunes-Quintanilla

**Affiliations:** 1 Facultad de Medicina Veterinaria y Zootecnia, Universidad Nacional Autónoma de México, Avenida Universidad 3000, Ciudad de México 04510, Mexico; 2 Departamento de Microbiología e Inmunología, Facultad de Medicina Veterinaria y Zootecnia, Universidad Nacional Autónoma de México, Avenida Universidad 3000, Ciudad de México 04510, Mexico; ninna_gr@hotmail.com; 3 Centro de Enseñanza, Investigación y Extensión en Ganadería Tropical, Facultad de Medicina Veterinaria y Zootecnia, Universidad Nacional Autónoma de México, Km. 5.5 Carretera Federal Tlapacoyan-Martínez de La Torre, Martínez de La Torre 93600, Mexico; alonsodm@unam.mx; 4 Facultad de Nutrición, Universidad Autónoma del Estado de Morelos, Calle Ixtaccíhuatl 100, Vista Hermosa, Cuernavaca 62350, Mexico; america.barrera@uaem.mx; 5 Centro Nacional de Investigación Disciplinaria en Salud Animal e Inocuidad—INIFAP, Carretera Federal Cuernavaca—Cuautla 8534, Col. Progreso, Jiutepec 62550, Mexico; reyes.david@inifap.gob.mx

**Keywords:** anti-tick vaccine, ADAMTSL, relative expression, *Rhipicephalus microplus*

## Abstract

The cattle tick, *Rhipicephalus microplus*, is the most significant ectoparasite in the cattle industry. The application of acaricides constitutes the main control method. However, inadequate treatments have serious drawbacks, including the appearance of multi-resistant ticks. Tick vaccines offer a safe and economically sustainable alternative for controlling *R. microplus*. Nevertheless, the efficacy of existing vaccines has been limited by polymorphisms in target antigens among strains from different geographical regions. In this study, we characterized a putative Metalloprotease from the ADAMTSL family. We analyzed three regions to evaluate their transcriptional profiling in different *R. microplus* tick tissues, using two constitutive genes (*β-tubulin* and *Elfa-1*) as references. The expression levels showed that ADAMTSL-R1 was upregulated 39.37-fold (*p* ≤ 0.05) in salivary glands. The ADAMTSL-R2 showed the highest expression, rising 7.69-fold (*p* ≤ 0.05) in ovaries and up to 59.39-fold (*p* ≤ 0.05) in egg mass. Furthermore, this region showed the highest level of conservation among *Rhipicephalus* isolates. The ADAMTSL-R3 was upregulated only in the egg mass. The results of this study provide a basis for future research focused on elucidating the role of these protein variants in tick biology, including their feeding mechanisms and potential implications in pathogen transmission. Understanding these factors may aid in developing an effective tick vaccine.

## 1. Introduction

The cattle tick, *Rhipicephalus microplus*, is a hematophagous arthropod distributed in the tropical and subtropical regions of Africa, Australia, and Latin America (32° N, 32° S). This species represents the most important challenge in the cattle industry, as it has the potential to transmit pathogens responsible for diseases such as babesiosis (*Babesia bovis* and *Babesia bigemina*) and anaplasmosis (*Anaplasma marginale*) [1]. The cattle industry is affected by decreased milk production, lower reproductive rates, and increased veterinary costs due to acaricide treatments. Additionally, intensive use, improper practices, and the genetic plasticity of *R. microplus* have led to resistance against most acaricide products, including organophosphates, pyrethroids, amidines, phenylpyrazolones, and macrocyclic lactones [2]. Globally, approximately one billion cattle are at risk of infestation by *R. microplus*, resulting in estimated annual economic losses ranging from USD 22 to 30 billion [3]. Alternative approaches have included using different cattle breeds, pasture management, plants as tick repellents or acaricides, microbial control, and anti-tick vaccines [4].

Anti-tick vaccines have become one of the most promising alternatives for managing *R. microplus* infestations, offering advantages such as specificity, environmental safety, improved animal and human health, easy administration, and cost reduction [5]. The recombinant Bm86 antigen is the only commercially available worldwide. However, its efficacy was limited due to genetic polymorphisms in the Bm86 sequence, which differed across geographic regions and tick populations [6]. Therefore, in recent years, methodological approaches have been studied to identify new targets for developing anti-tick vaccines using reverse vaccinology, which integrates functional genomics, *in silico* analysis, and systems biology [7]. The discovery of tick antigens against *R. microplus* has been particularly challenging due to the lack of a complete genome in databases. This is primarily attributed to its complexity, as the genome is approximately 7.1 Gb long, with 70% consisting of highly repetitive sequences, making its assembly exceptionally difficult [8]. However, transcriptomes from different tissues of adult *R. microplus* ticks, including ovaries [9], salivary glands, and larvae [10,11], have been reported. Recent studies have facilitated the identification of innovative vaccine antigen candidates. The evaluation of synthetic and recombinant proteins is currently underway to assess their potential to elicit protective immunity in cattle infested by *R. microplus* [7,12]. Furthermore, this approach has led to identifying target antigens (such as peptides and epitopes) that trigger the production of specific antibodies, which interfere with tick feeding [13,14]. Recently, it has been known that ticks have evolved mechanisms of evasion of the host immune response by producing a complex mixture of bioactive compounds (peptides/proteins, nucleic acids, and other molecules) through the tick’s salivary glands [15]. One of these proteins is metalloprotease, a multifunctional protein involved in various biological processes in vertebrates regulating physiological and pathological functions [16]. In ticks, these proteins are essential for embryogenesis and maintaining feeding-related functions, as they facilitate the degradation of fibrin and fibrinogen [17,18], enabling the tick to feed continuously from the host.

Metalloproteases have been studied, and some development has been undertaken as a tick vaccine against *R. microplus* infestations. Vaccination with rBrRm-MP4 demonstrated an overall efficacy of 60%, significantly reducing tick number, oviposition, and egg hatching, demonstrating its potential as a tick vaccine candidate [19]. Moreover, multicomponent vaccines combining recombinant antigens, such as Rm39, Rm76, Rm180, and Rm239, have achieved even higher efficacy, reducing *R. microplus* infestation by 73.2% in immunized cattle [20]. For this reason, the present work aimed to characterize a putative metalloprotease from the ADAMTSL (a disintegrin and metalloproteinase with thrombospondin type 1 repeats/motif-like) family using bioinformatics and functional transcriptomics approaches to evaluate the physicochemical and immunogenic properties. Subsequently, we identified three regions to evaluate the transcriptional profiling in different *R. microplus* tick tissues.

## 2. Materials and Methods

### 2.1. Ticks

The *R. microplus* (Susceptible “Media Joya”) adult female ticks and larvae were obtained from laboratory colonies kept at the Tick Laboratory of the National Center of Disciplinary Research in Animal Health and Safety from the National Institute for Forestry, Agricultural and Livestock Research (CENID-SAI, INIFAP) in Jiutepec, Morelos, Mexico. This tick strain was collected initially from cattle infested in Tapalpa, Jalisco, Mexico (19°56′41.0″ N 103°45′27.0″ W).

### 2.2. In Silico Analysis

An *in silico* analysis was conducted on the metalloprotease–disintegrin protein belonging to the ADAMTSL family. The genomic, coding DNA, and amino acid sequences were identified and retrieved from the National Center for Biotechnology Information (NCBI) database (GenBank accession no. ADK62391.1 and HM748961.1). A comprehensive bioinformatics workflow was employed to characterize the protein. Protein family and domain predictions were performed using UniProtKB (https://www.uniprot.org/ accessed on 1 February 2023) [21] and Interpro (https://www.ebi.ac.uk/interpro/ accessed on 1 February 2023) [22] to identify functional domains within the amino acid sequence. The physicochemical properties, including molecular weight, protein size, theoretical isoelectric point (PI), amino acid composition, half-life, instability index, aliphatic index, and hydrophobicity, were analyzed using ProtParam (https://web.expasy.org/protparam/ accessed on 15 February 2023) [23]. Secondary structure analysis was conducted with ProtScale (https://web.expasy.org/protscale/ accessed on 17 February 2023) [23], employing the Kyte and Doolittle algorithm to identify hydrophobic regions; values above 1.6 indicate hydrophobic residues, while values below 1.6 indicate hydrophilic residues. Transmembrane regions were predicted using TMHMM 2.0 (https://services.healthtech.dtu.dk/services/TMHMM-2.0/ accessed on 21 February 2023) [24]. The signal peptide was assessed with SignalP 6.0 (https://services.healthtech.dtu.dk/services/SignalP-6.0/ accessed on 21 February 2023) [25], and potential glycosylation sites were predicted with NetNGlyc-1.0 (https://services.healthtech.dtu.dk/services/NetNGlyc-1.0/ accessed on 24 February 2023) using the 0.5 glycosylation threshold [26]. The presence of linear B-cell epitopes was assessed using the following prediction tools: EMBOSS Antigenic (https://www.bioinformatics.nl/cgi-bin/emboss/antigenic accessed on 6 March 2023), considering an antigenic score ≥ 1.0 [27]; BepiPred-2.0 (http://www.cbs.dtu.dk/services/BepiPred/ accessed on 8 March 2023) using the 0.5 antigenicity threshold established by default [28]; and Predicting Antigenic Peptides (http://imed.med.ucm.es/Tools/antigenic.html accessed on 10 March 2023) using the Kolaskar and Tongaonkar algorithm with a score > 1.0. The overlapping amino acid sequences in the B-cell epitopes predicted by at least two tools were defined as the consensus predicted epitopes. Tertiary structure prediction was performed using the AlphaFold server (https://alphafoldserver.com/ accessed on 22 July 2024) [29] and SWISS-MODEL based on homology modeling (https://swissmodel.expasy.org/interactive accessed on 18 March 2024) [30]. Finally, molecular surface graphics of the hydrophobicity profiles, electrostatic potential distributions, and 3D model visualizations presented in this study were created using UCSF ChimeraX (https://www.rbvi.ucsf.edu/chimerax accessed on 3 April 2024) [31].

### 2.3. Sequence Analysis

Twenty-three nucleotide sequences were retrieved from GenBank for comparison with the metalloprotease–disintegrin sequence. The nucleotide and deduced amino acid sequences were analyzed using BioEdit Sequence Alignment Editor 7.2.5 for putative polymorphism. Further, amino acid sequences from each isolate were grouped according to identity/similarity using the SIAS tool (http://imed.med.ucm.es/Tools/sias.html accessed on 27 December 2024). Based on the information of the amino acid residues, it was investigated if there could be any putative antigenic change in the epitopes in the three regions selected, ADAMTSL-R1, ADAMTSL-R2, and ADAMTSL-R3, from available *Rhipicephalus* isolates using Jalview software version 2.11.4.1.

### 2.4. RNA Extraction and cDNA Synthesis

Five fully developed *R. microplus* (Susceptible “Media Joya”) female ticks were collected and washed thoroughly in PBS. The dorsal cuticle was dissected with a scalpel blade, and the salivary glands, midgut, and ovaries were separated using fine-tipped forceps, washed twice in PBS, and stored at −70 °C until used. Additionally, 100 mg of egg mass and unfed tick larvae were used for the experiment. Total RNA was extracted from the homogenized tick samples and pulverized in a frozen mortar using TRIzol reagent (Thermo Scientific^®^, Waltham, MA, USA) according to the manufacturer’s instructions. Briefly, 200 μL of chloroform were added to 1 mL of TRIZOL containing the homogenized samples, vortexed, and incubated for 5 min at room temperature (RT). The RNA-containing aqueous phase was centrifuged at 12,000× *g* for 10 min at 4 °C and transferred to a new tube. The RNA was precipitated by mixing an equal volume of isopropanol, and the pellets were obtained by centrifugation at 12,000× *g* for 10 min at 4 °C. The total RNA pellets were resuspended in diethylpyrocarbonate (DEPC)-treated water. The concentration was determined spectrophotometrically at 260 nm using a nanophotometer (Implen, Westlake Village, CA, USA), and the purity was assessed by the 260/280 nm optical density ratio. Only samples with ratios between 1.8 and 2.2 were included in subsequent analyses. Synthesis of cDNA was carried out from 5 μg of total RNA using RevertAid First Strand cDNA Synthesis (Thermo Scientific^®^, Waltham, MA, USA). The cDNA was subsequently used as a template to amplify the three regions selected from the metalloprotease–disintegrin protein.

### 2.5. RT-qPCR Assays

Primers were designed based on the immunogenic properties and functional domains of the metalloprotease–disintegrin protein from the *R. microplus*. This study identified three regions, ADAMTSL-R1, ADAMTSL-R2, and ADAMTSL-R3, along with the housekeeping genes *β-tubulin* and *Elfa-1*. The qPCR assays were performed in 0.2 mL nuclease-free tubes with a final volume of 20 μL per replication of each sample and analyzed in quadruplicate. The assays were prepared using commercial GoTaq^®^ qPCR Master Mix 2× (Promega, Madison, WI, USA) at a primer concentration of 20 μM. The amplification process was carried out using a Rotor-Gene thermocycler (Corbett 6000 Research, Qiagen, Hilden, Germany) with the following cycling conditions: an initial denaturation step at 95 °C for 5 min, followed by 40 cycles of denaturation at 95 °C for 10 s, alignment at 60 °C for 30 s, and extension at 72 °C for 30 s. Finally, the amplicons were analyzed by electrophoresis on 1% agarose gels stained with ethidium bromide and visualized under UV light using a Kodak Gel Logic 1500 Imaging System (Rochester, NY, USA).

### 2.6. Relative Expression

Relative expression analysis was estimated based on the cycle threshold values (Ct) per region and housekeeping gene. The larval stage was used as a control, and its gene expression was compared with that of the egg mass, midgut, ovaries, and salivary glands. The relative expression was performed using the 2^−(ΔΔCt)^ method with *β-tubulin* and *Elfa-1* as standardizing genes [32]. The data analysis was conducted using the Qiagen^®^ GeneGlobe Data Analysis Center platform (https://geneglobe.qiagen.com/mx/analyze accessed on 10 August 2023). The platform provided ΔCt, ΔΔCt, and fold change values for the groups of interest calculated as the ratio of the standardized expression of the interest group genes 2^−ΔCt^ to the standardized expression of the control group genes 2^−ΔCt^. The 2^−(ΔΔCt)^ values for each region and gene per group were automatically analyzed using Student’s *t*-test with a significance level of 0.05%.

## 3. Results

### 3.1. Bioinformatic Characterization

#### 3.1.1. Family and Functional Domains

The ADAMTSL protein sequence consists of 2727 amino acids, encoded by a coding sequence (CDS) of 8181 bp distributed across 40 exons. The analysis revealed that the protein belongs to the disintegrin and metalloproteinase with thrombospondin motif (ADAMTS/ADAMTS-like) family. In addition, the domain prediction resulted in conserved peptides identified in the following order: one ADAM-TS spacer 1 (130–244), six thrombospondin type-1 (TSP1) domains (253–312, 315–371, 411–460, 461–517, 524–586, 588–642), 10 pancreatic trypsin inhibitor Kunitz (Kunitz/BPTI) domains (1436–1486, 1495–1545, 1554–1604, 1613–1663, 1675–1725, 1733–1783, 1801–1851, 1858–1908, 1925–1975, 2002–2052), one WAP-type domain (2184–2237), three immunoglobulin I-set (Ig-like) domains (2281–2360, 2370–2442, 2502–2575), and one protease and lacunin (PLAC) domain (2583–2622).

#### 3.1.2. Physicochemical Analysis and Cellular Location and Structure

**Figure 1:**
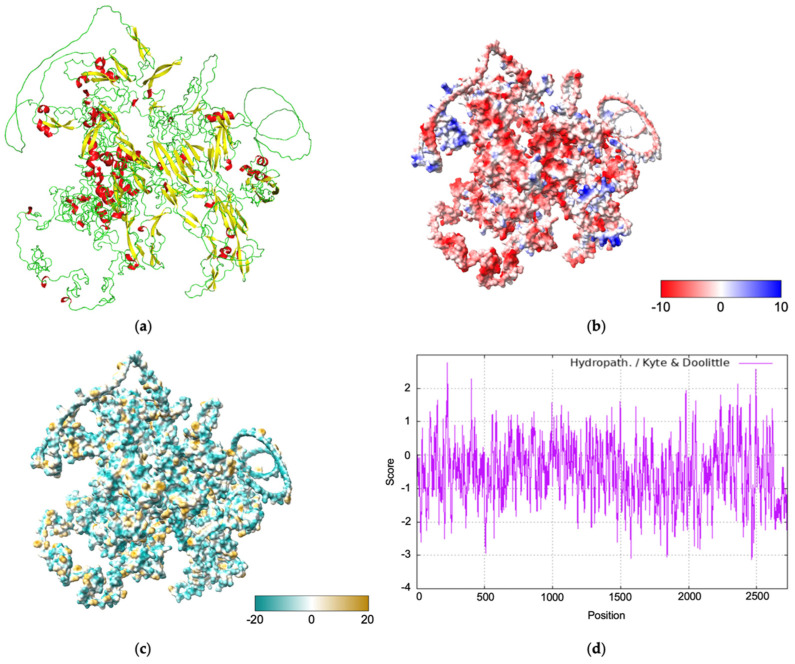
Structural *in silico* analysis. (**a**) The structural ensemble of the ADAMTSL (ADK62391.1) protein was predicted using the BetaAlphaFold 3 server. The color code indicates secondary structure: red (helix), yellow (sheet), and green (loop). (**b**) The electrostatic surface analysis was generated using the ChimeraX coulombic command. The surfaces are colored to represent electrostatic potential energy values, ranging from the lowest (red, −10) to the highest (blue, +10). (**c**) The hydrophobicity profile was generated using the ChimeraX mlp command. The surfaces indicate hydrophobic properties, ranging from hydrophilic (dark cyan, −20) to hydrophobic (gold, +20). (**d**) The Kyte and Doolittle hydropathy plot derived from the amino acid sequence of the ADAMTSL protein classifies regions as hydrophobic and hydrophilic.

The physicochemical analysis predicted a molecular weight of 295.8 kDa, a PI of 5, an instability index of 5, an aliphatic index of 49.59, and a hydropathy index of −0.507. The predicted half-life of the designed construct was 1.1 h (*in vitro* in mammalian reticulocytes), 3 min (*in vivo* in yeast), and 2 min (*in vivo* in *E. coli*). The cellular localization analysis revealed that ADAMTSL lacks a signal peptide and does not contain transmembrane regions. The tertiary structure prediction generated by AlphaFold 3 indicates that the protein is composed mainly of loops (green), followed by sheets (yellow), and, to a lesser extent, helices (red) (Figure 1a). The electrostatic potential analysis revealed a surface gradient transitioning from red (regions with a high negative charge) to blue (regions with a high positive charge), suggesting that the ADAMTSL protein surface mainly exhibits low electrostatic potential (Figure 1b). The structural surface analysis showed that the protein primarily consists of hydrophilic regions (dark cyan) (Figure 1c). This observation is corroborated by the hydropathy plot, generated using the Kyte and Doolittle algorithm, which indicates that over 85% of the amino acid residues have a value below 1.6 (Figure 1d).

#### 3.1.3. Immunogenic Analysis

**Figure 2:**
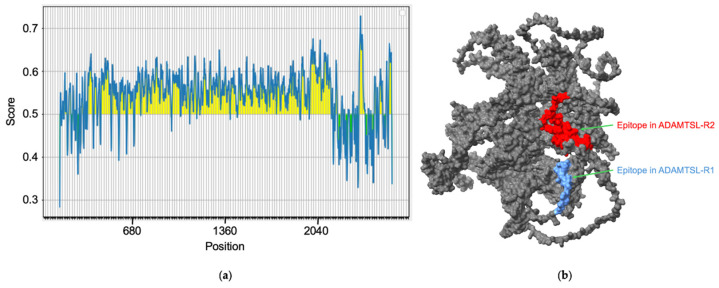
Prediction of antigenic regions through bioinformatic analysis. (**a**) Linear epitope prediction using BepiPred-2.0. Residues with scores above the threshold (0.5) are predicted to be part of an epitope and are colored yellow. (**b**) The surface regions colored blue show the epitope present in ADAMTSL-R1, while the regions in red show the epitope found in ADAMTSL-R2.

The immunogenic analysis revealed that the ADAMTSL protein contains potential linear epitopes throughout its amino acid sequence (Figure 2a). The sequences of the epitopes with the highest scores are shown in Table 1. Based on the expression levels and their location within the coding regions of the ADAMTSL gene, two epitopes were selected for their potential immunogenic properties and presence in regions of interest for expression analysis. The ADAMTSL-R1 region includes YRPVCRLVNKAGHCPVAEVEAQPAAVVRADCRDVCRVDADCPDIRK-CCYNGCAHVCVQAVVTEVTTVQTS, which is located in the WAP domain, while the ADAMTSL-R2 region includes SLELCKQRCAHVVAPAVVDTP, situated in the Kunitz/BPTI domain. Both analyzed epitopes are located on the surface of the ADAMTSL protein, making them accessible to immune system molecules (Figure 2b).

### 3.2. Analysis of the Metalloprotease–Disintegrin (ADAMTSL)

**Figure 3:**
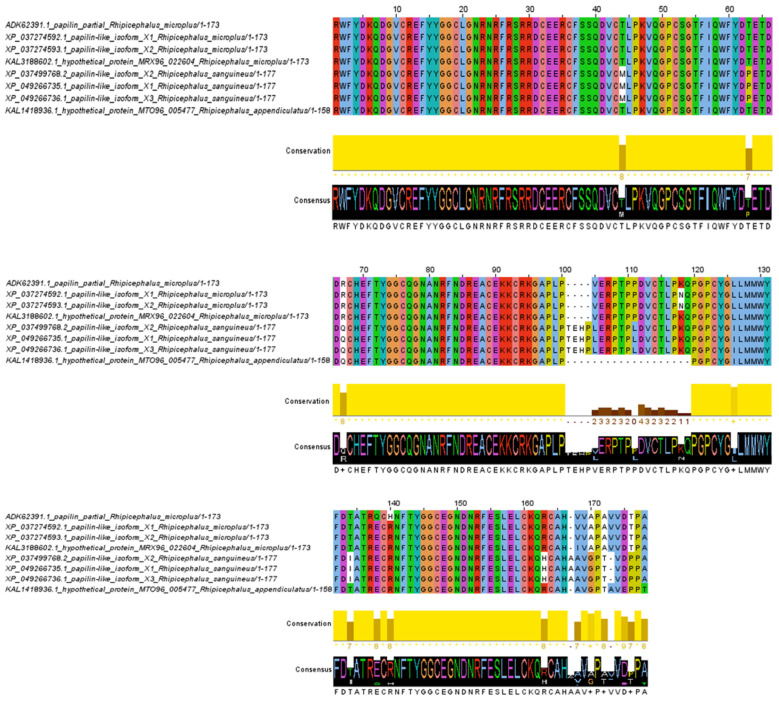
Amino acid alignment of ADAMTSL-R1 region. The sequences between *R. microplus*, *R. sanguineus*, and *R. appendiculatus* isolates were analyzed. Conservation plot scores show amino acid changes in specific positions in the protein region.

**Figure 4:**
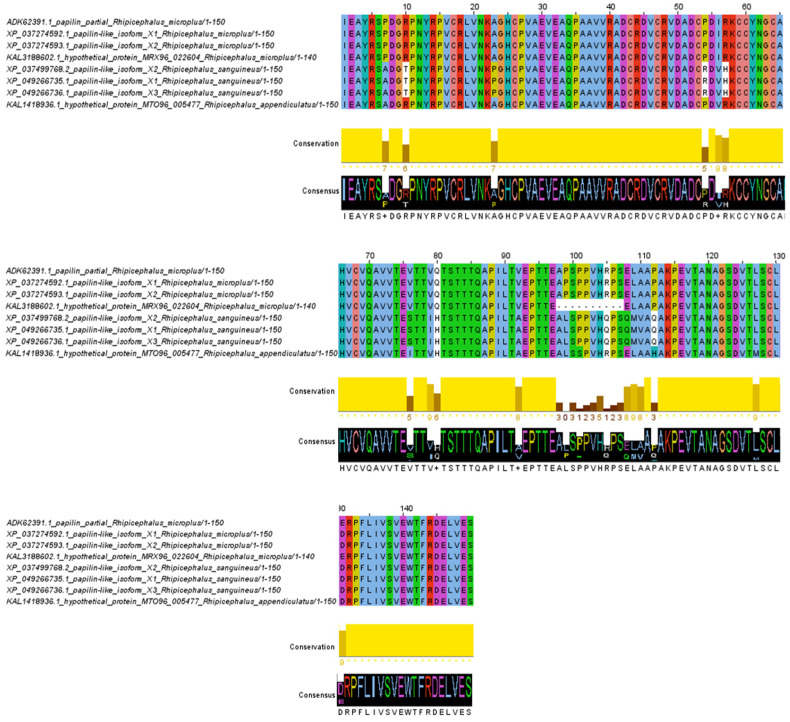
Amino acid alignment of ADAMTSL-R2 region. The sequences between *R. microplus*, *R. sanguineus*, and *R. appendiculatus* isolates were analyzed. Conservation plot scores show amino acid changes in specific positions in the protein region.

**Figure 5:**
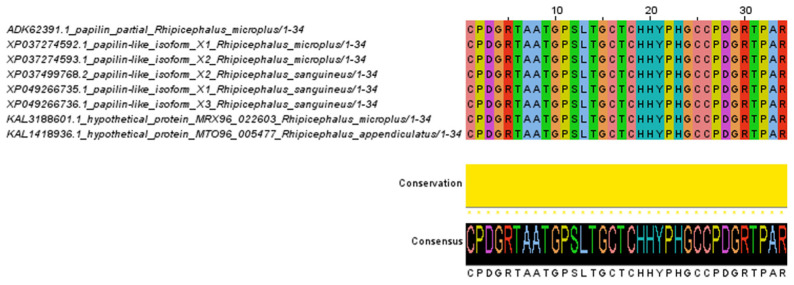
Amino acid alignment of ADAMTSL-R3 region. The sequences between *R. microplus*, *R. sanguineus*, and *R. appendiculatus* isolates were analyzed. Conservation plot scores show amino acid changes in specific positions in the protein region.

The amino acid alignment of the ADAMTSL-R1 region revealed 16 variable sites. Notably, the amino acid changes at positions 44, 63, 134, 163, and 167 are specific to the *R. sanguineus* isolates and are not found in the *R. microplus* and *R. appendiculatus* isolates. In contrast, the substitutions at positions 67, 126, and 170 are unique to *R. sanguineus* and *R. appendiculatus*, with no changes observed in the *R. microplus* isolates. Additionally, the *R. sanguineus* isolates contain four extra amino acids at positions 101–104 compared to the *R. microplus* isolates. Conversely, amino acids in positions 101–120 are absent from the isolates of *R. appendiculatus*. Interestingly, within the *R. microplus* isolates, only two amino acid changes were detected at positions 118 and 140 (Figure 3). In the ADAMTSL-R2 region, the amino acid alignment predicted 19 variable sites. The amino acid substitutions at positions 10, 23, 54, 57, 76, 79, 105, 108, 109, 110, and 112 are characteristic of the *R. sanguineus* isolates, distinguishing them from the *R. microplus* and *R. appendiculatus* isolates. In contrast, amino acid residues at positions 7, 80, and 92 are conserved in the *R. microplus* isolates, while the *R. sanguineus* and *R. appendiculatus* isolates share the same amino acid changes at these positions. Overall, the amino acid alignment of the ADAMTSL-R2 region shows a high level of conservation among isolates of *Rhipicephalus* species (Figure 4). No amino acid changes were observed in the ADAMTSL-R3 region (Figure 5).

### 3.3. Relative Expression

**Figure 6:**
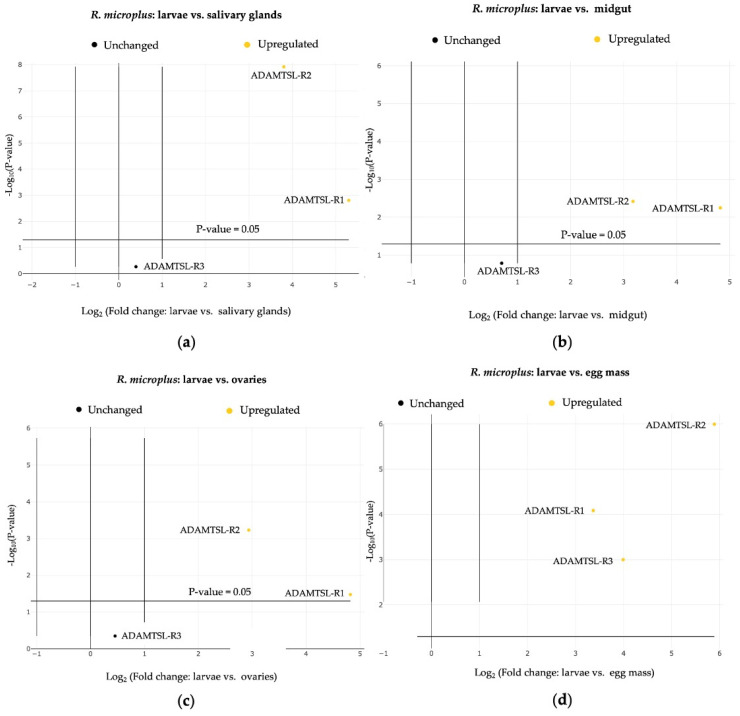
Relative transcript accumulation of ADAMTSL-R1, ADAMTSL-R2, and ADAMTSL-R3 in free life stages and adults’ tissue of *R. microplus.* The expression level of ADAMTSL showed variability among regions of interest. Differences were considered statistically significant when *p* < 0.05. (**a**) Control vs. salivary glands. (**b**) Control vs. midgut. (**c**) Control vs. ovaries. (**d**) Control vs. egg mass.

**Figure 7:**
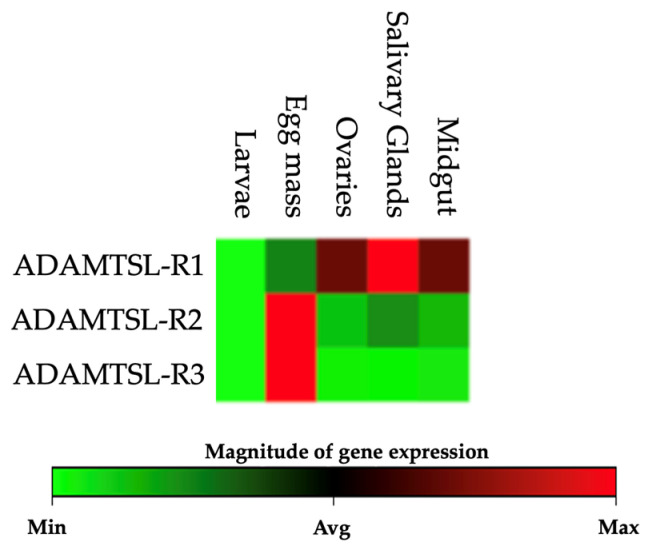
Expression heat map for each region in different *R. microplus* tick tissues. The color green represents a low expression level, and the red color represents a high expression level.

A comparative analysis of ADAMTSL-R1, ADAMTSL-R2, and ADAMTSL-R3 was conducted by comparing the relative expression values of each region. Significant differences (*p* ≤ 0.05) were evaluated among the tissues analyzed, including egg mass, larvae, salivary glands, midgut, and ovaries of *R. microplus* (Table 2). ADAMTSL-R2 exhibited the highest expression frequency across the tissues, increasing by 7.69-fold (*p* ≤ 0.05) in the ovaries and up to 59.39-fold (*p* ≤ 0.05) in the *R. microplus* egg mass. The ADAMTSL-R1 region showed an increase in expression levels in the salivary glands, reaching up to 39.37-fold (*p* ≤ 0.05). In general, both ADAMTSL-R1 and ADAMTSL-R2 were upregulated in all the tissues (Figure 6). In contrast, ADAMTSL-R3 was upregulated only in the egg mass. Non-significant (*p* > 0.05) overexpression values were observed for this region of salivary glands, midgut, and ovaries (Figure 7).

## 4. Discussion

Recent advancements in omics sciences have revolutionized vaccine development by incorporating bioinformatics tools for analyzing genomic, transcriptomic, and proteomic data, facilitating the identification of promising vaccine candidates. Research that integrates transcription profiling analyses with a reverse vaccinology approach offers a robust framework for recognizing novel prophylactic targets. This combination presents sustainable and efficient strategies for protecting cattle against tick infestations. Following this approach, bioinformatic characterization of a putative metalloprotease from the ADAMTSL family was performed. These are secreted extracellular matrix (ECM) regulatory proteins that play critical roles in tissue development, homeostasis, repair, and regeneration [33].

The identified ADAMTSL protein primarily comprises hydrophilic regions exposed to the extracellular space. No transmembrane or intracellular regions were detected, suggesting that ADAMTSL is secreted into the extracellular environment, similar to ECM proteins. This characteristic is important, as proteins secreted into the extracellular space are recognized as suitable antigens for vaccine development due to their accessibility to the host’s immune system [34,35,36]. The proteins secreted by ticks are crucial in pathogen transmission, feeding, and immune modulation. They are key components of tick biology and promising targets for developing novel vaccine antigens [37]. Moreover, no signal peptide was identified in the analyzed amino acid sequence, which may be due to the reported genetic sequence being “partially sequenced”, suggesting that the signal peptide could be present in the undetermined region of the protein.

In this study, bioinformatic analyses revealed the presence of linear B cell epitopes, suggesting that the ADAMTSL protein contains several regions capable of eliciting a strong immune response, which could be valuable for vaccine development targeting *R. microplus.* Furthermore, the desirable features for a vaccine candidate should include being encoded by a single gene, expressed across tick tissues and life cycle, playing a key role in tick biology, not sharing homology with the host *Bos taurus*, and being capable of stimulating both B and T cells to trigger an immune humoral response [38,39]. Our findings are promising, as this approach has demonstrated effectiveness in previous studies focused on immunizing cattle [40,41,42,43]. However, future validation of these epitopes through experimental methods will be required to confirm their immunogenicity and potential for protective immunity.

Upon identifying a candidate antigen for developing an anti-tick vaccine, it is important to produce a sufficient quantity of the antigen for subsequent validation processes. Depending on the antigen’s nature, biotechnological techniques should be employed to express the antigen in some host systems, such as bacteria, yeast, mammalian, or insect cells. Chemical synthesis may also be utilized. On the other hand, it is important to consider the immunogen formulation with carrier proteins or adjuvants as a part of the solution to enhance the immunogenicity, routes of administration, and immunization schedule. Once the experimental model for testing new antigens is established, the selected mammalian host must be immunized with the antigen, which is expected to generate a strong and long-lasting immune response [44].

The three-dimensional structure of the target protein revealed a model with 70.6% sequence identity to a protein from the tick *Ixodes ricinus* (GenBank accession no. A0A6B0VED9). This high sequence identity suggests a reliable structural homology that provides insights into potential molecular functions. The homologous protein is also associated with serine protease inhibition, playing key roles in blood feeding, blood flow regulation, host angiogenesis disruption, and wound healing [45,46]. The consistent conservation of the protein structure, particularly in the Kunitz/BPTI domains, across tick species, as supported by both predictive tools, highlights the likelihood of a similar functional role. Moreover, the shared presence of functional domains, including BPTI/Kunitz, WAP, Ig-like, and PLAC, further supports this hypothesis. These domains are known for their involvement in protease inhibition and host response modulation, essential for tick survival and pathogenicity [47]. Additionally, predictive analysis of N-glycosylation sites revealed the presence of 33 glycosylation sites distributed across the 2727 amino acids of the analyzed sequence. However, no N-glycosylation sites were detected in the ADAMTSL-R1, ADAMTSL-R2, and ADAMTSL-R3 regions. The role of glycosylation in tick vaccine antigens has not been fully elucidated, but it is suggested to play a significant role in influencing antigenicity, immunogenicity, and host immune recognition [48,49]. However, the absence of glycosylation sites may facilitate the expression of antigens (epitope-containing peptides) in bacterial recombinant expression systems, which are cost-effective, scalable, and easy to handle [50,51].

Interestingly, a comparative transcription profile analysis revealed that ADAMTSL-R1, which contains Kunitz/BPTI domains and an epitope with immunogenic characteristics, was overexpressed in the salivary glands; these findings are consistent with those reported by Tirloni [52], who identified increased transcripts levels of metalloproteases in the salivary structures of partially engorged *R. microplus* ticks. According to studies conducted by Dai [46] and Alim [53], the expression of mRNA-encoding proteins with Kunitz/BPTI domains is upregulated during blood-feeding processes in the host. In addition, these kinds of proteins have been extensively documented in tick species [11,54,55] and identified in the salivary glands of *R. microplus* ([52], this study), *I. scapularis* [17,56], *I. ricinus* [57], *I. persulcatus*, and *R. sanguineus* [58]. These findings suggest that the metalloprotease–disintegrin (ADAMTSL) plays a key role in the parasite-host interaction, facilitating successful blood feeding.

ADAMTSL-R2, which comprises WAP and Ig domains, was upregulated in the egg mass and showed the highest level of conservation among the *Rhipicephalus* isolates. Firstly, this upregulation may be attributed to the role of certain metalloproteases in mediating interactions with embryonic morphogenetic proteins and their involvement in energy metabolism during embryogenesis [59,60,61]. In addition, it is posited that secreted proteins may contribute to the immune response during the embryonic development of *R. microplus* ticks [62]. Secondly, as discussed previously, it is essential to study proteins in full detail, analyzing the genetic variability from tick species in specific geographical locations to avoid polymorphisms directly affecting the development of anti-tick vaccines [63,64]. Finally, ADAMTSL-R3 was upregulated exclusively in the egg mass, which supports the hypothesis that this protein plays a key role during embryogenesis, regulating ADAMTS protease activity and ECM remodeling [65].

Overall, the comparative analysis of transcription profiles revealed that ADAMTSL-R1, ADAMTSL-R2, and ADAMTSL-R3 were overexpressed at varying levels across the analyzed tissues. This observation can be explained by the documented occurrence of alternative splicing events in ADAMTSL family proteins, including papilin in *Caenorhabditis elegans* [66] and *Drosophila melanogaster* [67]. Moreover, it has been hypothesized that a specific alternative splicing region may lead to the loss or gain of Kunitz/BPTI and Ig-like domains [68,69]. According to Kramerova [67], ADAMTSL proteins, such as papilin, are critical for extracellular matrix organization, cellular rearrangement, and the modulation of metalloprotease activity during organogenesis. In fact, RNAi-mediated suppression of papilin expression in *C. elegans* leads to alterations in basal membrane formation and embryonic lethality, suggesting that papilin is important for nematode embryogenesis [66]. In arthropods, RNAi disruption in *Nilaparvata lugens* causes phenotypic defects, such as egg non-hatching and abnormal embryo development [70].

## 5. Conclusions

This is the first report to provide bioinformatic characterization, immunological analysis, and transcriptional profiling of three specific regions of a protein belonging to the ADAMTSL family in different *R. microplus* tick tissues. These findings indicate that ADAMTSL-R1, ADAMTSL-R2, and ADAMTSL-R3 show significantly differing expression levels throughout the egg mass and larval stages, as well as in adult tissues, including the salivary glands, midgut, and ovaries of *R. microplus*. Furthermore, analyses of the ADAMTSL-R2 region showed a significant level of conservation among *Rhipicephalus* isolates, demonstrating the presence of epitopes within the putative metalloprotease ADAMTSL protein. In addition, the presence of immunogenic regions within ADAMTSL proteins highlights their potential as candidates for anti-tick vaccines. However, additional research is required to investigate the genetic and physiological diversity among tick isolates from several geographical regions and across tick species; this knowledge would be important to develop an effective anti-tick vaccine. Finally, targeting these proteins could interfere with tick development and reproduction, offering a sustainable and effective alternative to controlling *R. microplus* infestations.

## 6. Patents

The work presented in this manuscript is part of an ongoing patent application.

## Figures and Tables

**Table 1:** Epitopes identified in aminoacidic sequence of ADAMTSL protein.

Residue	Epitope Sequence	Domain
2184–2237	* YRPVCRLVNKAGHCPVAEVEAQPAAVVRADCRDVCRVDADCPDIRKCCYNGCAHVCV**Q**AVVTEVTTVQTS	WAP
523–641	ESTKECVV**D**AVCNGT	TSP1
1927–1976	* SLELCKQRCAHVVA**P**AVVDTP	Kunitz_BPTI
2473–2573	GNEVVT**V**DVVIAT	Ig-like
2363–2451	HQKVSHVTNLVV**Y**VPATIRPSSATVTVTVSE	Ig-like
1493–1546	PDACLL**P**KVVGPCDGQ	Kunitz/BPTI
222–232	ETILI**V**LLYQE	N/A
1343–1357	GCECQNLVY**G**CCPDG	N/A
2140–2167	DHRG**C**VQCRCSNPCETFSCHEAELCRIE	N/A
998–1002	EGCCV**V**TEFGCCRDN	N/A
2569–2590	NQSVS**V**HIVMDDIHVPESCTDS	Ig-like
1242–1261	EGCICSRLLY**G**CCPDDVTPA	N/A
410–460	IREVVC**I**NP	TSP1

Residues with the highest score are shown in bold. * Indicates the sequences analyzed for their relative expression.

**Table 2:** Comparative expression of different regions of ADAMTSL gene in tissues of *Rhipicephalus microplus*.

Tissue	Relative Expression		
	ADAMTSL-R1	ADAMTSL-R2	ADAMTSL-R3
Egg mass	10.32 *	59.32 *	15.89 *
Salivary glands	39.37 *	13.97 *	1.32
Midgut	28.26 *	9.05 *	1.63
Ovaries	28.26 *	7.65 *	1.37

* *p* < 0.05; bold letter: upregulated; italic letter: sub-regulated; black: normalized.

## Data Availability

The databases analyzed during the current study are available from the corresponding author upon reasonable request.
